# A high-density genetic map and QTL analysis of agronomic traits in foxtail millet [*Setaria italica* (L.) P. Beauv.] using RAD-seq

**DOI:** 10.1371/journal.pone.0179717

**Published:** 2017-06-23

**Authors:** Jun Wang, Zhilan Wang, Xiaofen Du, Huiqing Yang, Fang Han, Yuanhuai Han, Feng Yuan, Linyi Zhang, Shuzhong Peng, Erhu Guo

**Affiliations:** 1 Millet Research Institute, Shanxi Academy of Agricultural Sciences, Changzhi, Shanxi, China; 2 Shanxi Key Laboratory of Genetic Resources and Breeding in Minor Crops, Changzhi, Shanxi, China; 3 Research Institute of Agriculture Sciences of Yanan, Yanan, Shaanxi, China; 4 Shanxi Agricultural University, Taigu, Shanxi, China; National Institute of Plant Genome Research, INDIA

## Abstract

Foxtail millet (*Setaria italica*), a very important grain crop in China, has become a new model plant for cereal crops and biofuel grasses. Although its reference genome sequence was released recently, quantitative trait loci (QTLs) controlling complex agronomic traits remains limited. The development of massively parallel genotyping methods and next-generation sequencing technologies provides an excellent opportunity for developing single-nucleotide polymorphisms (SNPs) for linkage map construction and QTL analysis of complex quantitative traits. In this study, a high-throughput and cost-effective RAD-seq approach was employed to generate a high-density genetic map for foxtail millet. A total of 2,668,587 SNP loci were detected according to the reference genome sequence; meanwhile, 9,968 SNP markers were used to genotype 124 F_2_ progenies derived from the cross between Hongmiaozhangu and Changnong35; a high-density genetic map spanning 1648.8 cM, with an average distance of 0.17 cM between adjacent markers was constructed; 11 major QTLs for eight agronomic traits were identified; five co-dominant DNA markers were developed. These findings will be of value for the identification of candidate genes and marker-assisted selection in foxtail millet.

## Introduction

Foxtail millet (*Setaria italica*) is one of the oldest cereals in the world, and is thought to have been domesticated from the wild species green foxtail, more than 8,000 years ago in northern China [1]. Foxtail millet has many excellent characteristics, among which C_4_ photosynthesis, which is the primary mode of carbon capture for some of the world’s most important food, feed, and fuel crops, such as maize, sorghum, sugarcane and switchgrass [2]. In addition, foxtail millet is known for its nutritional value: its grains have high protein, folic acid, vitamin E, carotenoids, and selenium [3–5]. In recent years, foxtail millet has become a valuable model for investigating plant architecture, drought tolerance, and C_4_ photosynthesis of grain and bioenergy crops because of its small genome size, self-fertilization, and short growth cycle [6–8]. Therefore, it is essential to assess its agronomic traits by developing a genetic linkage map and identifying genes or quantitative trait loci (QTLs).

Genome mapping of foxtail millet using molecular markers started in the 1990s, and a map including 160 restriction fragment length polymorphism (RFLP) markers was constructed in an intervarietal cross [9]. Later, Jia et al. [10, 11] developed 30 expressed sequence tag (EST)-derived simple sequence repeats (SSR) in foxtail millet, and constructed an integrated map with 81 SSR markers and 20 RFLP markers. By exploiting EST sequences, Gupta et al. [12] reported 98 potential intron length polymorphic (ILP) markers. However, these are low-throughput molecular markers that limit the efficiency and accuracy of QTL mapping.

The assembled reference genome of foxtail millet was released in 2012 by two independent groups [13, 14]. The availability of the foxtail millet genome sequence to the public provides an important resource for crop genetics and breeding. On the basis of the reference genome, a large number of markers (SSRs, EST-SSRs, SNPs, InDels, SVs, and TEs) have been developed and utilized [15–21]. For example, Pandey et al. [19] scanned the whole genome sequence of foxtail millet and identified a total of 28,342 microsatellite repeat motifs (SSRs); these markers showed a high percentage (~90%) of cross-genera transferability across millets, including green foxtail, cereals and bioenergy grasses. In previous studies, some traits were analyzed in foxtail millet. Doust et al. [22] identified 25 QTLs for vegetative branching. Meanwhile, Wang et al. [23] detected five QTLs related to plant height, panicle length, panicle weight, and grain weight. Sato et al. [24] analyzed and mapped the *stb1* gene, which is responsible for the trait of “spikelet-tipped bristles”. Mauro-Herrera et al. [25] analyzed the differences in flowering time under varying environmental conditions and identified 18 QTLs. Qie et al. [26] detected 18 QTLs for germination and early seeding drought tolerance. Fang et al. [16] described 29 QTLs responsible for 11 agronomic and yield traits. Moreover, *SiDWARF2*, *SiYGL1*, and *SiAGO1b* were fine mapped and cloned using dwarf mutant, AGO1 mutant, and yellow-green leaf mutant by map-based cloning [27–29]. Recently, multiple essential agronomic and quality traits have been studied by identifying QTLs or genes using NGS technologies in foxtail millet. Jia et al. [30] sequenced 916 foxtail millet varieties and identified 512 loci associated with 47 agronomic traits using genome-wide association studies (GWAS). Bai et al. [15] re-sequenced the foxtail millet landrace ‘Shi-Li-Xiang’ (SLX) and finely mapped a waxy gene using newly developed DNA markers. Masumot et al. [31] carried out QTL-seq and rapidly mapped the *NEKODE1* gene responsible for tip-branched panicle.

Indeed, most agronomic traits are determined by QTLs [32–34]. It is important to rapidly identify each locus or the major locus of QTLs for efficient crop breeding by marker-assisted selection (MAS). However, whole-genome deep re-sequencing remains cost-prohibitive for sequencing and genotyping of large populations, and is generally unnecessary. Restriction site-associated DNA sequencing (RAD-seq) reduces genome complexity by sequencing only the DNA fragments with restriction sites regardless of length, and is considered a useful tool for SNP discovery and genetic mapping [35–37].

In this study, a high-throughput and cost-effective RAD-seq approach was employed to generate a high-density genetic map for foxtail millet. The characteristics of this genetic map were analyzed and discussed in detail below. Moreover, 11 major QTLs were identified for eight distinct agronomic traits.

## Materials and methods

### Plant materials

Two foxtail millet cultivars, Hongmiaozhangu (P_1_) and Changnong35 (P_2_) were selected as female and male parents, respectively, for the mapping population. Hongmiaozhangu is characterized by low plant height, multiple tillers, and a small panicle. Changnong35 is characterized by high plant height, no tillering, and large panicle. The mapping parents were crossed at the Millet Research Institute (Changzhi, Shanxi) in 2013, and three real F_1_ hybrids were obtained. F_2_ seeds were obtained from a self-pollinated F_1_ individual in 2014. In 2015, the parents and 124 F_2_ plants obtained from one F_1_ individual were planted, and plant height (PH, cm), main panicle length (MPL, cm), main panicle diameter (MPD, cm), first main internode diameter (FMID, cm), second main internode diameter (SMID, cm), and third main internode diameter (TMID, cm) were assessed in the mature stage. Main panicle weight per plant (MPWP, g) and main grain weight per plant (MGWP, g) were measured after harvest. Data were analyzed using SPSS 17. Genomic DNA was extracted from young leaf tissues of parental and 124 F_2_ plants using a Plant DNA Kit (OMEGA, USA, D3485-02).

### RAD-seq of the parental lines and F_2_ population

We employed the RAD protocol described by Baird et al. [35]. The enzymes and restriction fragment sizes were evaluated based on the reference genome sequence (https://www.ncbi.nlm.nih.gov/genome/?term=foxtail+millet). *Tap*I was selected for RAD library construction. The library for Illumina sequencing was constructed from 200 ng of each DNA sample. All library were sequenced using Illumina HiSeq X Ten at Shanghai Major Biological Medicine Technology Co., Ltd.

### SNP identification and genotyping

For SNP calling, the Burrows-Wheeler Aligner [38] was applied for sequence alignment between the individual reads and the reference genome sequence, the Genome Analysis ToolKit [39] was used to detect SNP loci, and SAMtools [40] was used to filter out SNP loci. In this study, filtering of SNP loci was based on three criteria: (i) average sequence depth is < 5-fold in parents and < 3-fold in the progeny; (ii) no polymorphism between the parents; (iii) heterozygous in parents.

### Development of new DNA markers

Primers were designed according to the flanking sequences (300 bp upstream and 300 bp downstream of the selected SNPs). The primer sequences used for the new DNA markers are listed in S4 Table. PCR was carried out in a 10 μL volume containing 40 ng genomic DNA, 1.0 μL 10× reaction buffer, 0.2 μL 10 mmol L^–1^ dNTPs, 1.0 μL primer, 1 U rTaq DNA polymerase (TaKaRa, Dalian), and 5.7 μL ddH_2_O. The PCR was performed by initially denaturing the template DNA at 94°C for 5 min, followed by 35 cycles at 94°C for 30 s, 58°C for 30 s, and 72°C for 30 s, and then terminated by a final extension for 10 min at 72°C. PCR fragments were separated on 8% non-denatured polyacrylamide gel electrophoresis (PAGE) and visualized by silver staining [41].

### Linkage map construction

The poorly performing markers were removed before map construction, which excessively missed with more than 30% missing data in the F_2_ population. Markers with significant segregation distortion (χ^2^ test, P < 0.05) were excluded from the subsequent linkage map construction. Construction of the linkage map was performed using MSTmap software [42]. The major parameters for loci partition were as follows: distance_function for Haldane; p_value for 0.0000001; no_map_dist for 10; missing_threshold for 0.3; objective_function for Maximum likelihood. A total of 10,016 SNP markers were considered for linkage map construction. LOD = 10 was used to partition the SNP markers into linkage groups. Linkage groups from the same chromosome were merged together, and SNP markers of the same chromosome were again reordered with MSTmap.

### QTL analysis

QTL analysis was conducted using CIM (composite interval mapping) of the R/qtl package [43]. LOD thresholds for testing the significance of QTL peaks of each trait were calculated using 1,000 permutations, with a confidence interval of at least 80%. The position and effect of significant QTL was assessed for additive effects and percentage phenotypic variation explained (PVE%) by fitting a model containing all QTL identified for a given trait in R/qtl. The method of naming QTLs was as follows: *q* plus trait abbreviation and chromosome number; plus -1, -2, etc., when multiple QTLs for one trait were detected on the same chromosome.

## Results and discussion

### Phenotypic data of eight agronomic traits

Phenotypic data of the eight agronomic traits were analyzed (Table 1 and S1 Fig). A wide range of variation was observed in the eight agronomic traits in the F_2_ population; the absolute value of the skewness and kurtosis of most traits was less than 1, indicating that these traits had approximately normal distribution. The correlation among the eight traits were calculated. In previous study, Wang et al. [23] found significant associations of PH and MPL with PWP and GWP, respectively. Fang et al. [16] suggested that MPL, MPD, PWP, and GWP are positively correlated with one another. Similar results were reported by Wang et al. [44]. In the present study, all eight traits showed positive correlations with one another, except PH which had non-significant correlations with MPD, FMID, SMID, and TMID (Table 2), corroborating the results of previous studies.

**Table 1:** Phenotypic data analyses of eight agronomic traits for 124 F_2_ individuals.

Trait	P_1_	P_2_	Population				
			Mean	Max	Min	Skewness	Kurtosis
PH (cm)	154.0	176.8	185.5	214.0	113.5	-1.60	5.39
MPL (cm)	21.7	19.4	24.3	34.6	11.8	-0.23	1.07
MPD (cm)	1.8	3.1	1.8	5.3	3.2	0.23	-0.56
FMID (cm)	0.6	0.9	1.0	1.5	0.5	0.16	0.48
SMID (cm)	0.5	0.9	1.0	1.5	0.5	0.10	0.52
TMID (cm)	0.5	0.8	0.9	1.6	0.5	0.66	1.91
MPWP (g)	11.6	28.3	37.0	77.2	10.5	0.55	0.95
MGWP (g)	10.0	27.1	29.8	60.7	3.9	0.10	0.68

**Table 2:** Correlation coefficients among agronomic traits in 124 F_2_ individuals.

Traits	PH	MPL	MPD	FMID	SMID	TMID	MPWP
MPL	0.488**						
MPD	-0.088	0.355**					
FMID	0.064	0.400**	0.548**				
SMID	0.052	0.346**	0.516**	0.916**			
TMID	0.011	0.310**	0.542**	0.848**	0.897**		
MPWP	0.223*	0.462**	0.678**	0.610**	0.622**	0.623**	
MGWP	0.238**	0.389**	0.595**	0.530**	0.543**	0.551**	0.973**

*, ** Correlation is significant at the probability levels of 0.05 and 0.01, respectively plant height (PH, cm), main panicle length (MPL, cm), main panicle diameter (MPD, cm), first main internode diameter (FMID, cm), second main internode diameter (SMID, cm), third main internode diameter (FMID, cm), Main panicle weight per plant (MPWP), main grain weight per plant (MGWP)

### RAD-seq analysis and SNP identification in parental lines and F_2_ individuals

High-throughput genotyping by sequencing is an option for efficient marker-assisted breeding [45]. Currently, there are many new methods using NGS for identifying genes or QTLs, including GWAS [46], QTL-seq [47], MutMap [48], RAD-seq [35], and SLAF-seq [49], and some have been applied to foxtail millet [15, 30, 31]. However, the utility of RAD-seq has yet not been reported. RAD-seq sequences short DNA fragments with restriction sites digested by restriction endonucleases, regardless of length. It has been applied for SNP identification and linkage map construction in various organisms, including barley, snail, and ryegrass [50–53].

In the present study, a total of 830,740,674 reads were obtained for the parental lines and F_2_ population. After removal of low-quality reads, 760,378,099 high-quality reads were obtained, representing 91.5% of all reads. The high-quality reads were subsequently mapped to the reference genomic sequence, with a mapping ratio of at least 82% (S1 Table).

A total of 2,668,587 SNP loci were detected, including 2,629,567 located on nine chromosomes and the remaining 39,020 found on scaffolds. Among the 2,629,567 SNP loci, 97.5% (2,564,566) were filtered because of low sequence depth and lack of polymorphism between parents. The remaining 65,001 SNP loci were further screened to remove markers unsuitable for genetic map construction.

Among the 65,001 SNP markers, the numbers of markers on the chromosomes ranged from 4,414 to 10,625; the markers covered at least 98.37% of the physical length of the genome (Table 3). The marker density along each chromosome ranged from 104.82 to 261.63 markers per Mb, averaging 164.64 markers per Mb. The highest marker density (261.63/Mb) was found on chromosome 8, followed by chromosome 7 (213.19/Mb); the lowest marker density (104.82/Mb) was found on chromosome 1.

**Table 3:** Number and coverage of SNP markers on the nine chromosomes.

Chr.	Marker	Cover length (Mb)	Chr. Length (Mb)	Coverage (%)	Density (marker/Mb)
1	4414	42.11	42.15	99.92	104.82
2	7592	49.14	49.20	99.87	154.51
3	8017	50.65	50.65	100.00	158.28
4	5323	40.38	40.41	99.93	131.82
5	6274	47.08	47.25	99.63	133.27
6	6048	35.97	36.01	99.87	168.15
7	7542	35.38	35.96	98.37	213.19
8	10625	40.61	40.69	99.81	261.63
9	9166	58.72	58.97	99.57	156.10
Total	65001	400.03	401.30	99.66	164.64

**Fig 1:**
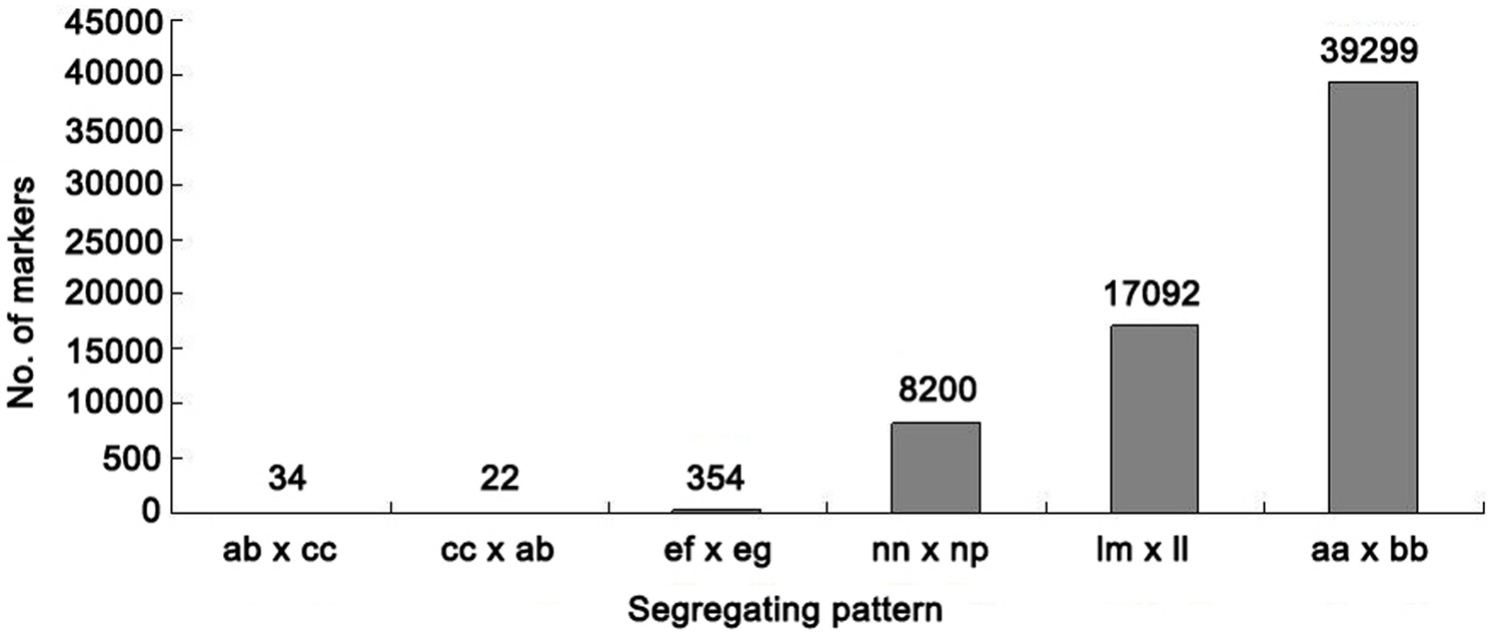
Number of markers for each segregation pattern.

In this study, we genotyped the parental lines using different letters and determined the segregation patterns of the mapping population. The genotypes of the SNP loci were encoded according to the paternal and maternal genotypes instead of the reference sequence. One locus with a homozygous SNP in the paternal and maternal genotypes would be encoded as aa × bb; if a certain SNP was heterozygous for one or two parents, the genotypes of the markers for the two parents were encoded as, for example, cc × ab or ef × eg. In total, we successfully coded 65,001 polymorphic SNP loci. Furthermore, these SNPs were classified into six segregation patterns (ab × cc, cc × ab, ef × eg, nn × np, lm × ll, and aa × bb). Finally, according to the two parent genotypes, 39,299 markers, which fell into the aa × bb segregation pattern, were used in linkage analysis (Fig 1).

### High-density genetic linkage map with SNPs

**Fig 2:**
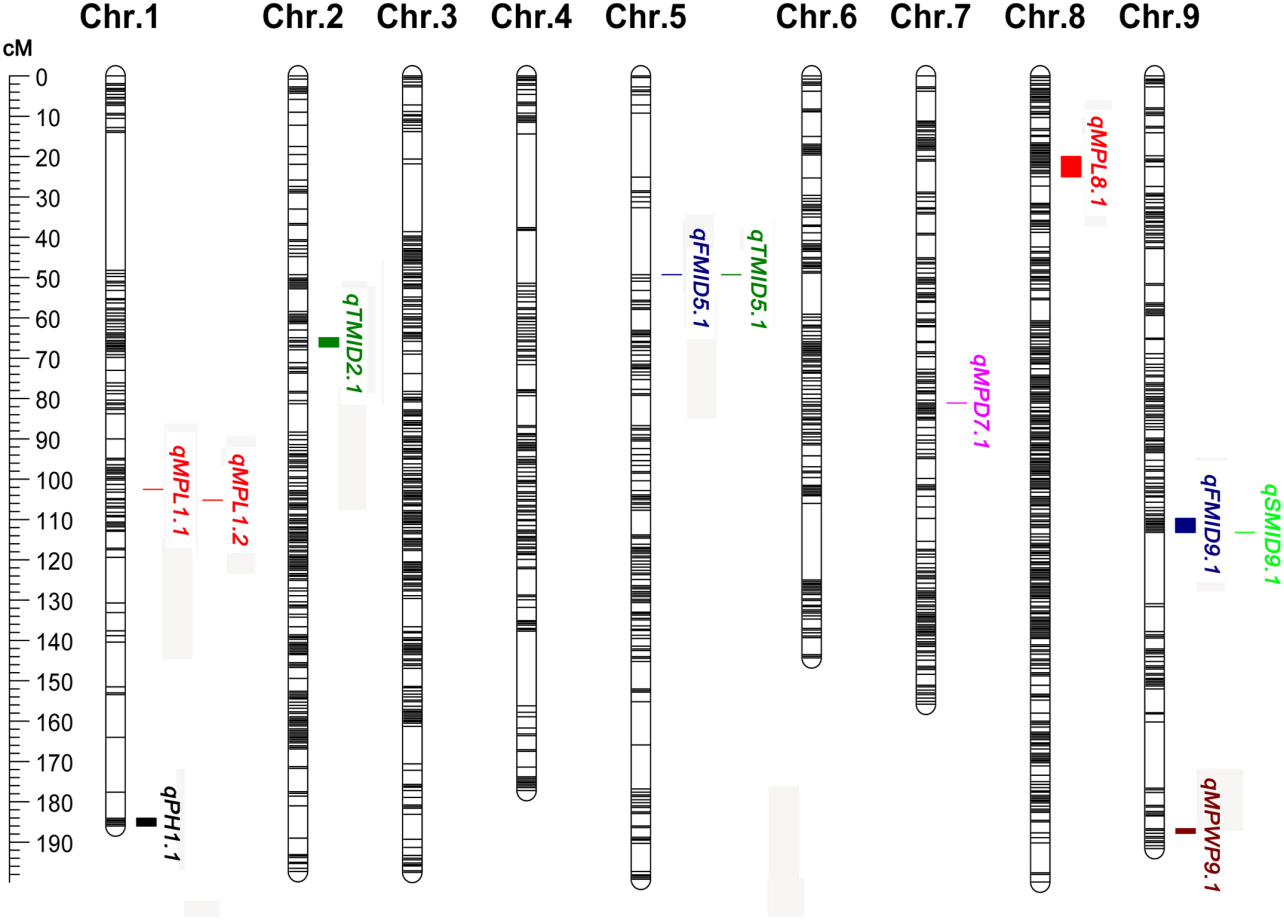
Genetic linkage map and QTLs controlling agronomic traits.

Genetic linkage maps play a major role in clarifying the genetic control of important traits. In particular, high-density molecular markers can be used to quickly map agronomic traits and to identify candidate genes within a region of interest [54]. In this study, after removing incomplete (26,906), significant segregation distortion (24,706), and non-aa × bb (3.373) SNP markers, 10,016 SNP markers were retained for genetic map construction. Finally, a high-density genetic map was constructed containing a total of 9,968 SNP markers. The markers were grouped into nine linkage groups and ordered (Fig 2 and S2 Table). The total genetic distance of the generated map was 1648.8 cM, with an average distance of 0.17 cM between adjacent markers. The largest linkage group was Chr. 8 with 2541 SNP markers and a length of 199.9 cM; the smallest was Chr. 6 with 841 SNP markers and a length of 144.3 cM. Two large gaps (>20 cM) were identified on Chr. 1 (34.23 cM) and Chr. 4 (23.21 cM), respectively. The highest missing data was found on Chr. 6 with 8.42%; the lowest missing data (6.52%) was found on Chr. 7 (Table 4).

**Table 4:** Characteristics of the high-density genetic map.

Linkage group	No. of markers	Distance (cM)	Average distance between markers (cM)	Largest gap	Missing data (%)
Chr. 1	369	186.0	0.50	34.23	7.80%
Chr. 2	1373	197.3	0.14	8.00	8.36%
Chr. 3	1553	197.5	0.13	16.80	7.58%
Chr. 4	597	177.2	0.30	23.21	7.16%
Chr. 5	605	199.2	0.33	16.65	6.69%
Chr. 6	841	144.3	0.17	18.95	8.42%
Chr. 7	1212	155.8	0.13	7.77	6.52%
Chr. 8	2541	199.9	0.08	7.41	7.24%
Chr. 9	877	191.6	0.22	17.70	7.96%
Total	9968	1648.8	0.17	34.23	8.42%

SNP markers are efficient for high-density genetic map construction since they allow high-throughput assessment, compared to RFLP and SSR markers. Currently, the most saturated intervarietal map was constructed by Fang et al. [16]. Compared with this map, the number of mapped loci (1035 SSR makers vs. 9968 SNP markers), marker density (26.25 marker/Mb vs. 164.64 marker/Mb), average distance between adjacent markers (1.27 cM vs. 0.17 cM) and total map length (1318.8 cM vs 1648.8 cM) were significantly increased in the newly constructed SNP maker genetic map. The marker number (9,968 SNPs) in this map was also higher than that of the two consensus maps constructed by Zhang et al. [14] (118 SNPs) and Bennetzen et al. [13] (992 SNPs). The current map provides not only a large number of SNP markers for foxtail millet, but also useful data for QTL analysis, gene fine mapping, and molecular breeding.

**Fig 3:**
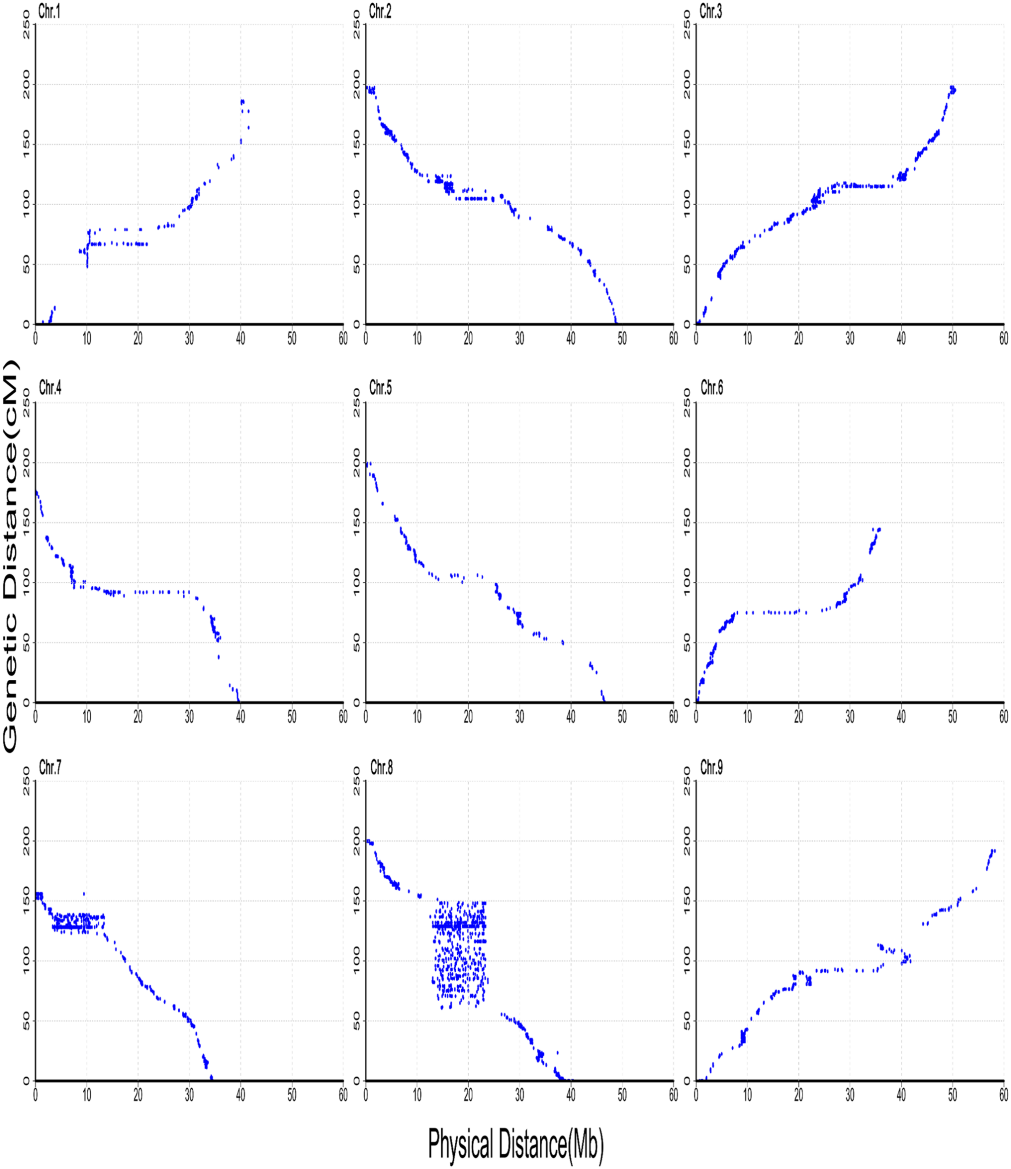
Genetic distance vs. physical distance for 9,968 SNPs in foxtail millet.

The collinearity of each chromosome with the reference genome was also analyzed (Fig 3). The average ratios of genetic-to-physical distance in low- and high-recombination chromosomes were 3.3 cM/Mb (Chr. 9) and 4.98 cM/Mb (Chr. 8), respectively (S3 Table). The 9,968 SNP markers in the genetic map covered 393.53 Mb of the physical length, spanning approximately 76.4% of the foxtail millet genome (∼515 Mb).

In this study, high levels of collinearity for each chromosome were revealed compared with the reference genome. A relatively low collinearity was observed between Chr. 8 and the reference genome. Non-collinearity could result from multiple factors, including intra- and inter-chromosomal locus duplication, genome rearrangement, transposon-mediated marker transposition, discrepancy in recombination rate among different genomic regions, small mapping population size, compromised marker ordering in the consensus map, missing data, and genotyping errors [55–57].

### QTLs for agronomic traits

Agronomic traits play an important role in the breeding of crops. The more QTLs of agronomic traits that are identified, the more they promote breeding by MAS. Recently, Chinese scientists have reported the successful development of new elite varieties in rice by pyramiding major genes that significantly contribute to grain quality and yield from three parents over 5 years, and demonstrated that rational design is a powerful strategy for meeting the challenges of future crop breeding [58]. In foxtail millet, QTLs controlling agronomic traits have been detected in previous studies [16, 22, 23, 30]. Wang et al. [23] and Fang et al. [16] reported QTLs for PH, MPL, MPD, PWP, and GWP, using F_2_ populations and SSR markers. Jia et al. [30] assessed a natural population of foxtail millet and SNP markers under five different environmental conditions, and identified 512 loci associated with 47 agronomic traits. In the present study, with the exception of MGWP, a total of 11 QTLs were identified for eight agronomic traits using the F_2_ population and SNP markers (Table 5, Fig 2). The 11 QTLs were mapped to Chr. 1, Chr. 2, Chr. 5, Chr. 7, Chr. 8, and Chr. 9.

**Table 5:** QTLs controlling agronomic traits in the Hongmiaozhangu × Changnong35 F_2_ population.

Trait	QTL	Chr.	P	LOD-threshold	LOD	Position (cM)	Marker number	PVE (%)	Additive effect
PH (cm)	*qPH1*.*1*	1	0.2	3.10	3.36	184.84	19	11.1	6.03
MPL (cm)	*qMPL1*.*1*	1	0.1	3.47	3.48	102.48	11	11.5	1.44
	*qMPL1*.*2*	1	0.1	3.47	3.57	105.15	5	11.8	1.36
	*qMPL8*.*1*	8	0.2	3.11	3.66	20.39	65	12.1	1.63
MPD (cm)	*qMPD7*.*1*	7	0.1	3.60	3.85	81.12	16	12.6	-0.15
FMID (cm)	*qFMID9*.*1*	9	0.05	3.98	4.80	110.86	47	15.5	0.10
	*qFMID5*.*1*	5	0.1	3.59	3.67	49.33	1	12.1	-0.09
SMID (cm)	*qSMID9*.*1*	9	0.05	3.97	4.11	113.15	3	13.5	0.09
TMID (cm)	*qTMID2*.*1*	2	0.2	3.19	3.30	64.86	22	11.0	0.08
	*qTMID5*.*1*	5	0.2	3.19	3.26	49.33	1	10.8	-0.09
MPWP (g)	*qMPWP9*.*1*	9	0.1	3.64	3.92	187.83	10	12.9	-2.48

MPL had three QTLs, the most prominent of which was designated *qMPL8*.*1*, that explained 12.1% of the phenotypic variance. Sixty-five SNP markers covered this interval. The other two QTLs (*qMPL1*.*1* and *qMPL1*.*2*) were detected on Chr. 1 with LOD scores of 3.48 and 3.57, and explained 23.3% of the phenotypic variance. Of these, *qMPL1*.*1* and *qMPL1*.*2* have also been detected in the haplotype and SSR maps [16, 30]. Two QTLs were detected for FMID, with the largest effect displayed by *qFMID9*.*1*, which explained 15.5% of the phenotypic variance. Forty-seven SNP markers were identified within the chromosomal region of *qFMID9*.*1*. Two QTLs were detected for TMID, with the largest effect displayed by *qTMID2*.*1*, which explained 11.0% of the phenotypic variance. A total of twenty-two SNP markers were found within the chromosomal region of *qTMID2*.*1*. The other QTL, *qTMID5*.*1* with 10.8% of the phenotypic variance was also detected in the haplotype map [30].

PH, MPD, and MPWP had only one QTL each, with LOD scores of 3.36, 3.85, and 3.92, and explained 11.1%, 12.6%, and 12.9% of the phenotypic variance, respectively. *qFMID5*.*1* and *qTMID5*.*1* shared the same chromosomal region, with LOD scores of 3.67 and 3.26, and explained 12.1% and 10.8% of the phenotypic variance, respectively.

With the exception of *qMPL1*.*1* and *qTMID5*.*1*, the remaining nine QTLs were identified for the first time, indicating that differences in QTL number and position might be attributed to different mapping populations (genotypes, population sizes, etc.), type and number of markers (i.e., RFLPs, SSRs, and SNPs), and environmental effects.

The additive effects of *qPH1*.*1*, *qMPL1*.*1*, *qMPL1*.*2*, *qMPL8*.*1*, *qFMID9*.*1*, *qSMID9*.*1*, and *qTMID2*.*1* were derived mainly from the female parent (Hongmiaozhangu), whereas those of *qMPD7*.*1*, *qFMID5*.*1*, *qTMID5*.*1*, and *qMPWP9*.*1* stemmed mainly from the male parent (Changnong35).

### Newly developed DNA markers

**Fig 4:**
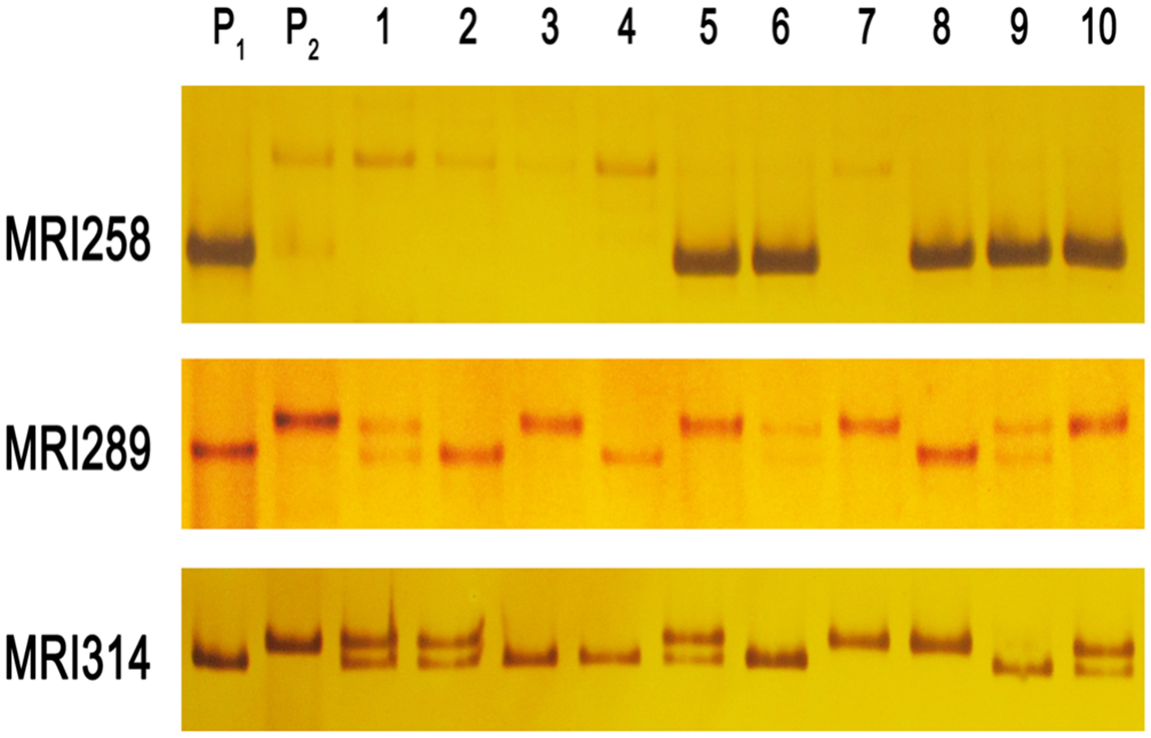
The amplification of newly developed DNA markers in the parents and F_2_ individuals.

In order to utilize the SNPs identified between Hongmiaozhangu and Changnong35, five SNPs were randomly selected in the intervals of identified QTLs. Based on the flanking sequences of selected SNPs, we designed primers and amplified the target sequences by PCR using genomic DNA of the two parents and their progenies. Finally, five co-dominant DNA markers were developed (Fig 4 and S4 Table). These markers will be useful for gene cloning and molecular breeding of foxtail millet.

### Mapping population

The construction of F_2_ populations is general straightforward, and F_2_ populations provide abundant information suitable for gene or QTL mapping and genetic analysis for many qualitative and quantitative traits. To date, there have been many attempts to use F_2_ populations directly for QTL analysis in crops. For example, QTLs were obtained that were associated with: crown rust susceptibility in ryegrass; drought-induced flag leaf senescence in wheat; awn, incomplete panicle exertion, and total spikelet number in rice; and aluminum-toxicity tolerance in soybean [32, 59–61]. In this study, the two parental lines have contrasting values across a wide range of agronomic traits, i.e. PH (154.0 and 176.8), MPD (1.8 and 3.1) (Table 1), which are essential for QTL identification. Finally, 11 major QTLs (PVE% > 10) were identified for eight agronomic traits. However, QTLs are sensitive to different environmental factors, such as years, regions, and to sometimes even materials [30, 62]. Therefore, different QTLs were more likely to be obtained in various studies. In particular, in the F_2_ population, it is important to verify the identified QTLs because of the lack of repeats. In future experiments, we will construct an recombinant inbred line (RIL) population to repeatedly verify the QTLs identified in the present study.

### Conclusions

Using a high-throughput and cost-effective RAD-seq approach, we developed a total of 9,968 SNPs to construct a high-density genetic linkage map for foxtail millet, spanning 1648.8 cM, with an average distance of 0.17 cM between adjacent markers. In total, 11 major QTLs were identified for eight agronomic traits, and nine QTLs (*qPH1*.*1*, *qMPL1*.*2*, *qMPL8*.*1*, *qFMID9*.*1*, *qSMID9*.*1*, *qTMID2*.*1*, *qFMID5*.*1*, *qMPD7*.*1*, and *qMPWP9*.*1*) were newly identified. Moreover, five co-dominant markers were developed based on the SNPs between the two parents in the region of the identified QTLs. Our results lay an important foundation for candidate gene identification and MAS breeding of foxtail millet.

### Accession number

Raw sequence data obtained in this study have been deposited in the NCBI Sequence Read Archive (SRA) with accession number SRP102319.

## Supporting information

S1 Fig Phenotypic frequency distribution of eight agronomic traits in Hongmiaozhangu × Changnong35 124 F_2_ individuals. (TIF)

S1 Table The sequencing summary of the parental lines and F_2_ individuals. (XLS)

S2 Table 9,968 SNP markers in the genetic map. The genotypes of the 124 individuals were also included.
(XLSX)

S3 Table Genetic and physical distances among 9,968 SNP markers in foxtail millet. (XLS)

S4 Table List of primers of newly developed DNA markers. (XLSX)

## Abbreviations

EST: expressed sequence tag
FMID: first main internode diameter
ILP: intron length polymorphic
MAS: marker-assisted selection
MGWP: main grain weight per plant
MPD: main panicle diameter
MPL: main panicle length
MPWP: main panicle weight per plant
NGS: next-generation sequencing
PH: plant height
QTL: quantitative trait locus
RAD-seq: restriction site-associated DNA sequencing
RFLP: restriction fragment length polymorphism
SMID: second main internode diameter
SNP: single-nucleotide polymorphism
TMID: third main internode diameter
