# Supplementary figures and images for: A high-density genetic map and QTL analysis of agronomic traits in foxtail millet [Setaria italica (L.) P. Beauv.] using RAD-seq
Source: PLoS One. 2017 Jun 23;12(6):e0179717. doi: 10.1371/journal.pone.0179717 (PMC5482450; doi:10.1371/journal.pone.0179717)

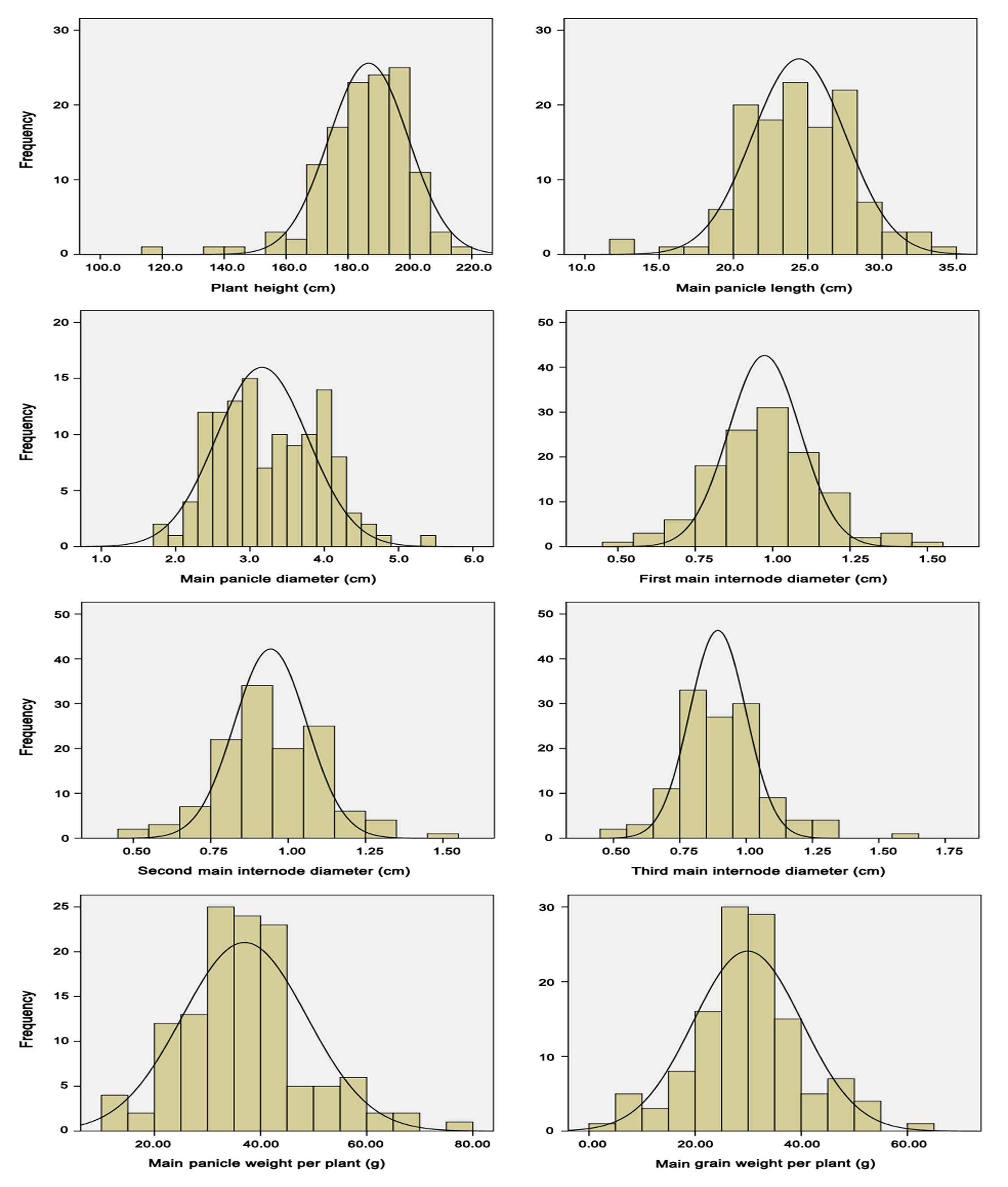

Supplement: S1 Fig — (TIF) [file pone.0179717.s001.tif]
